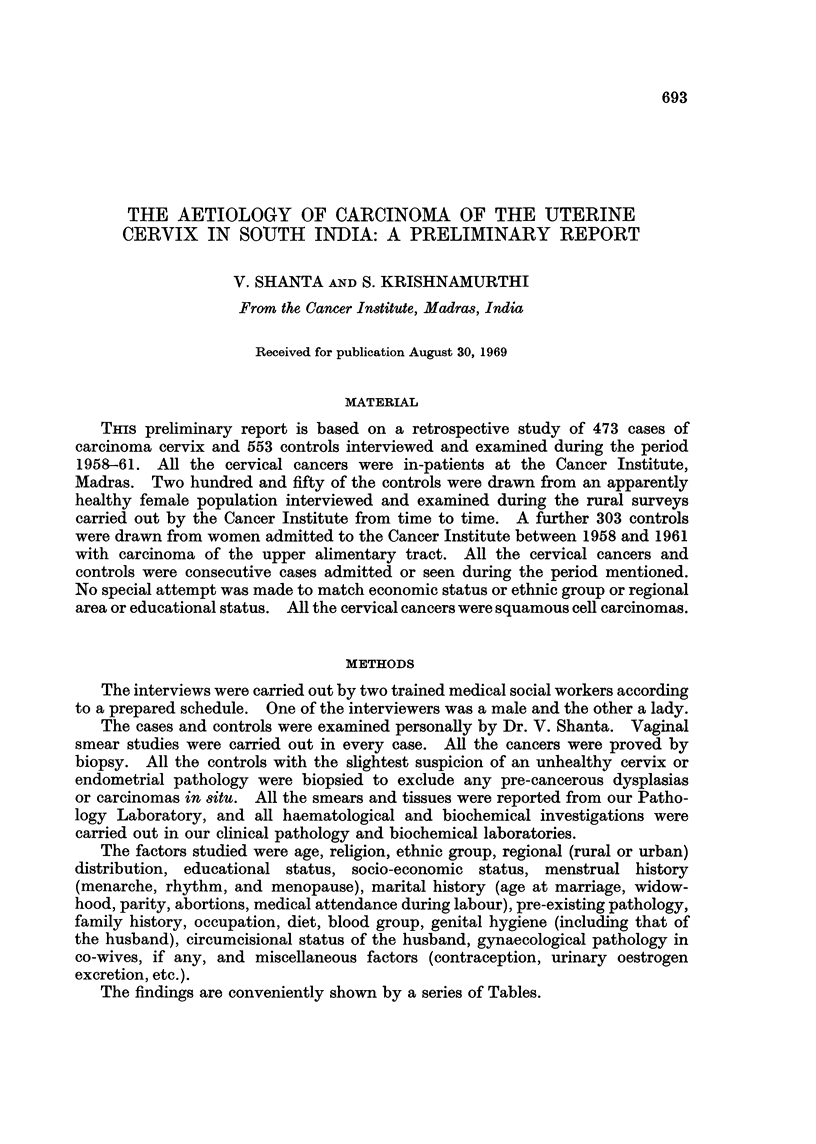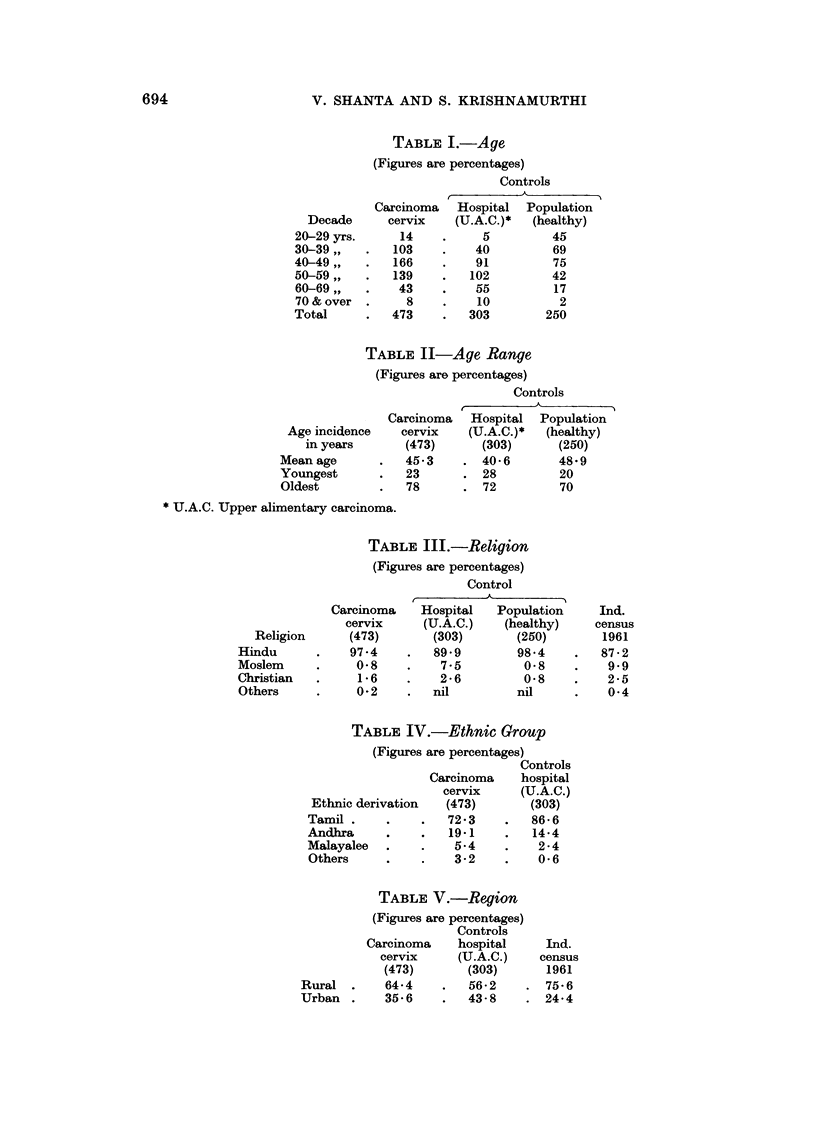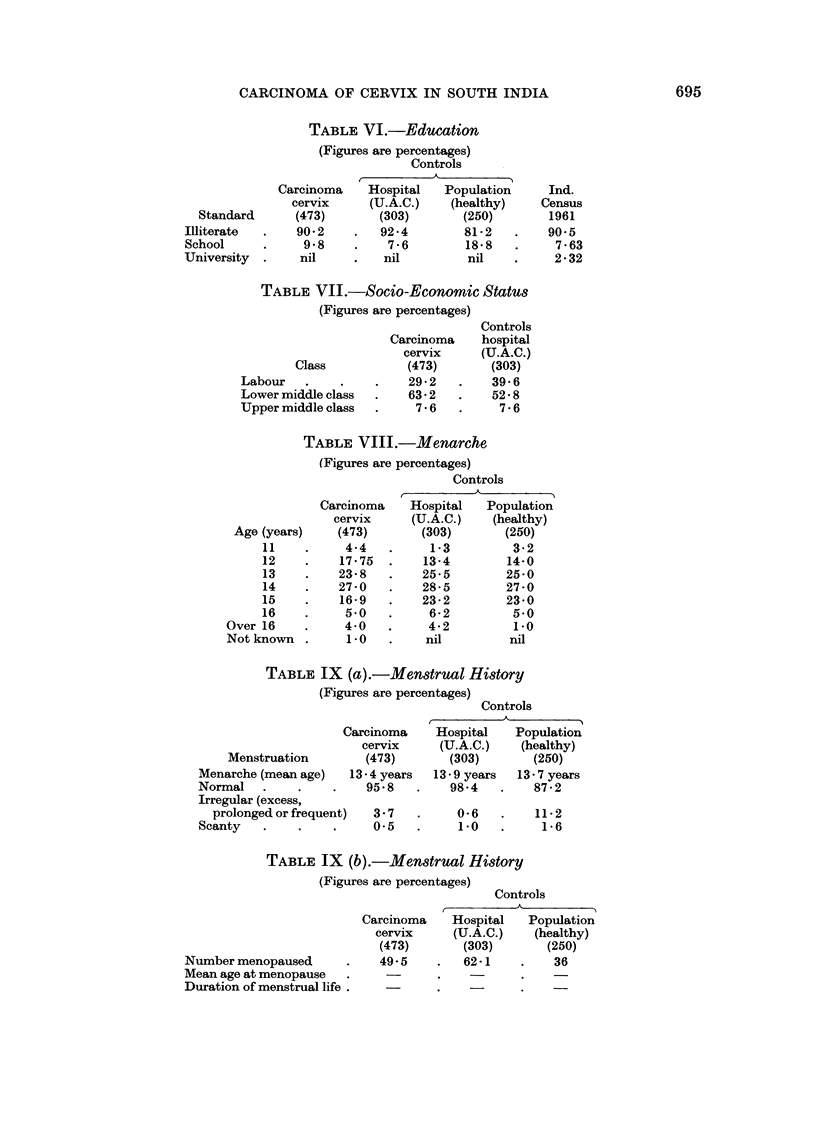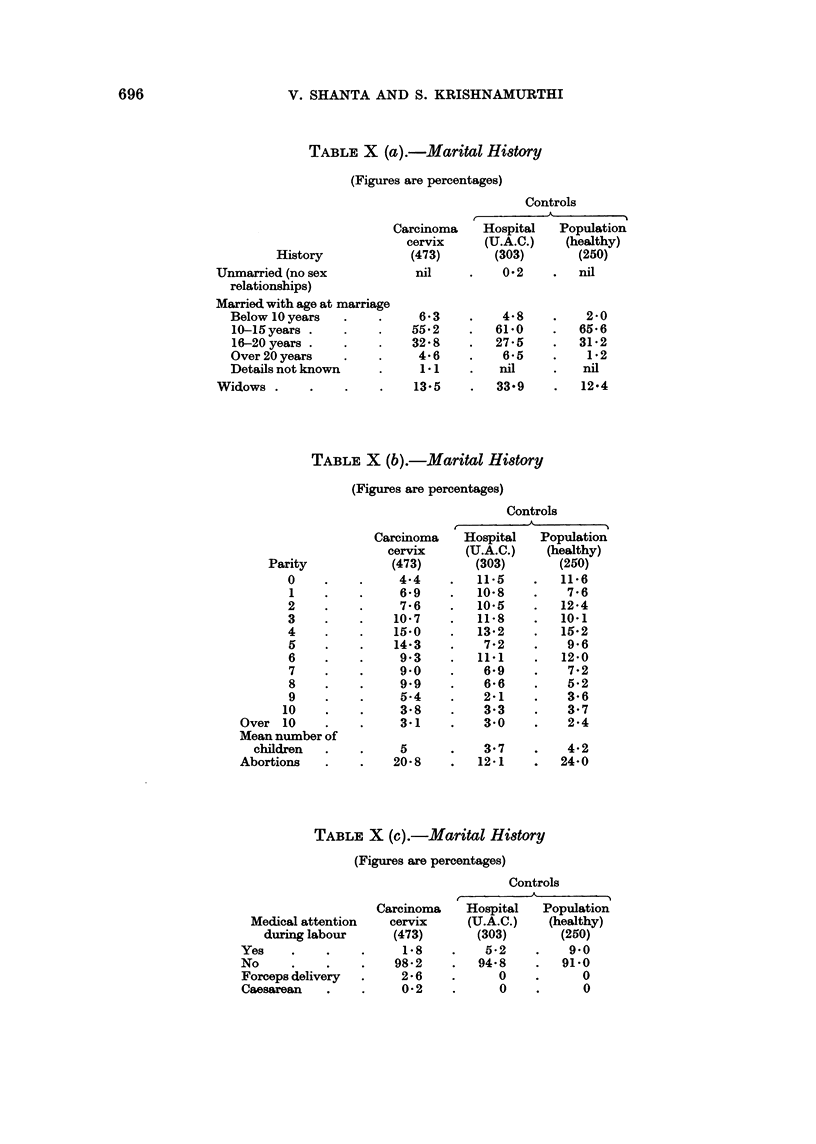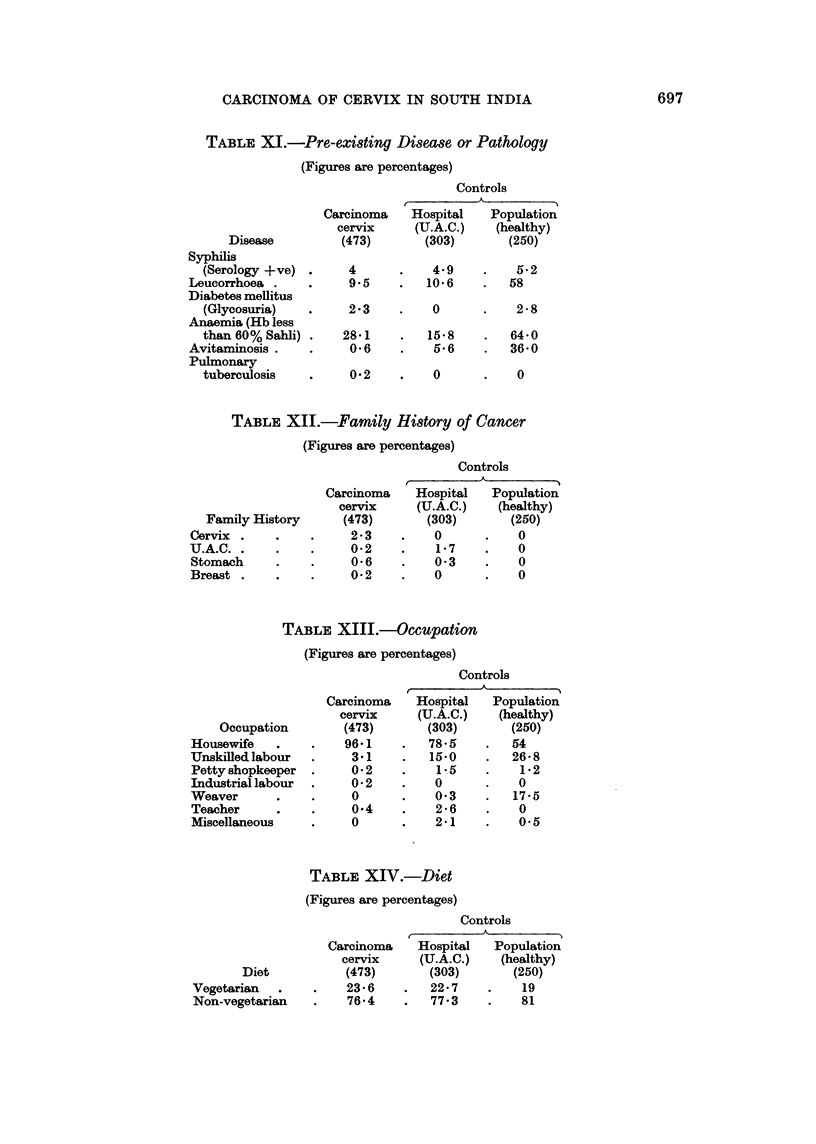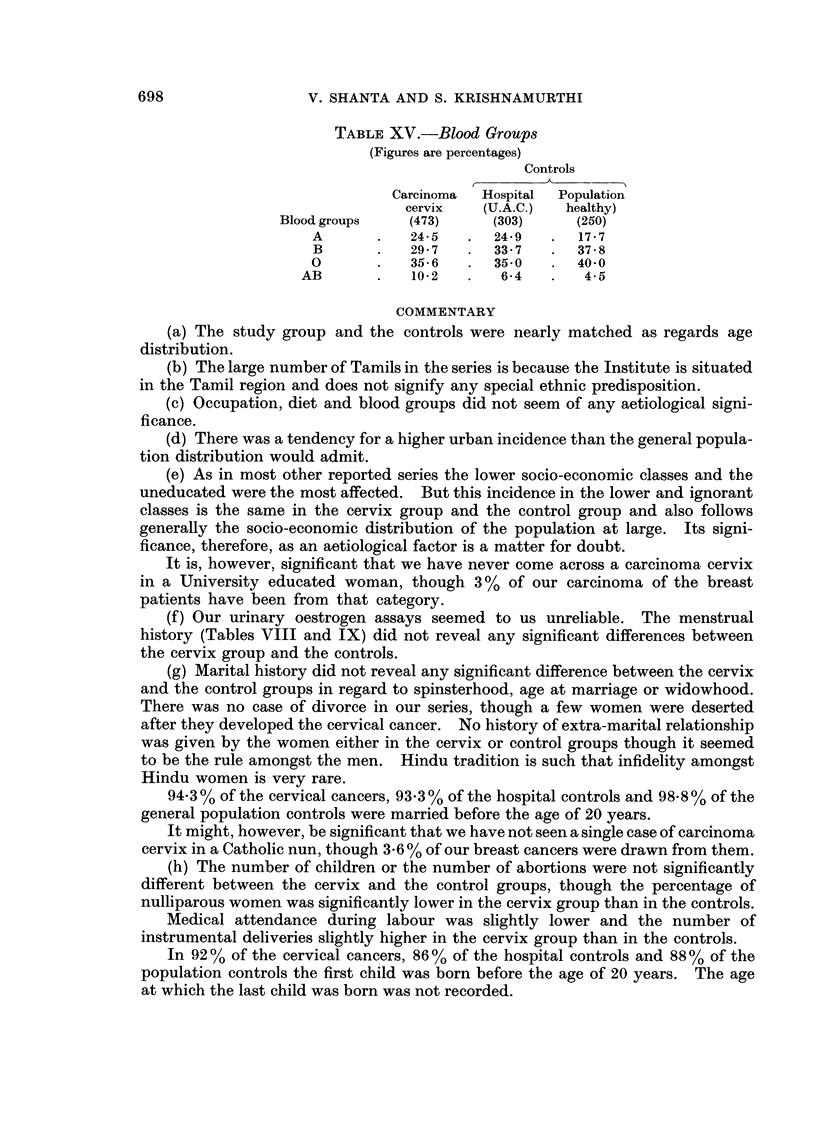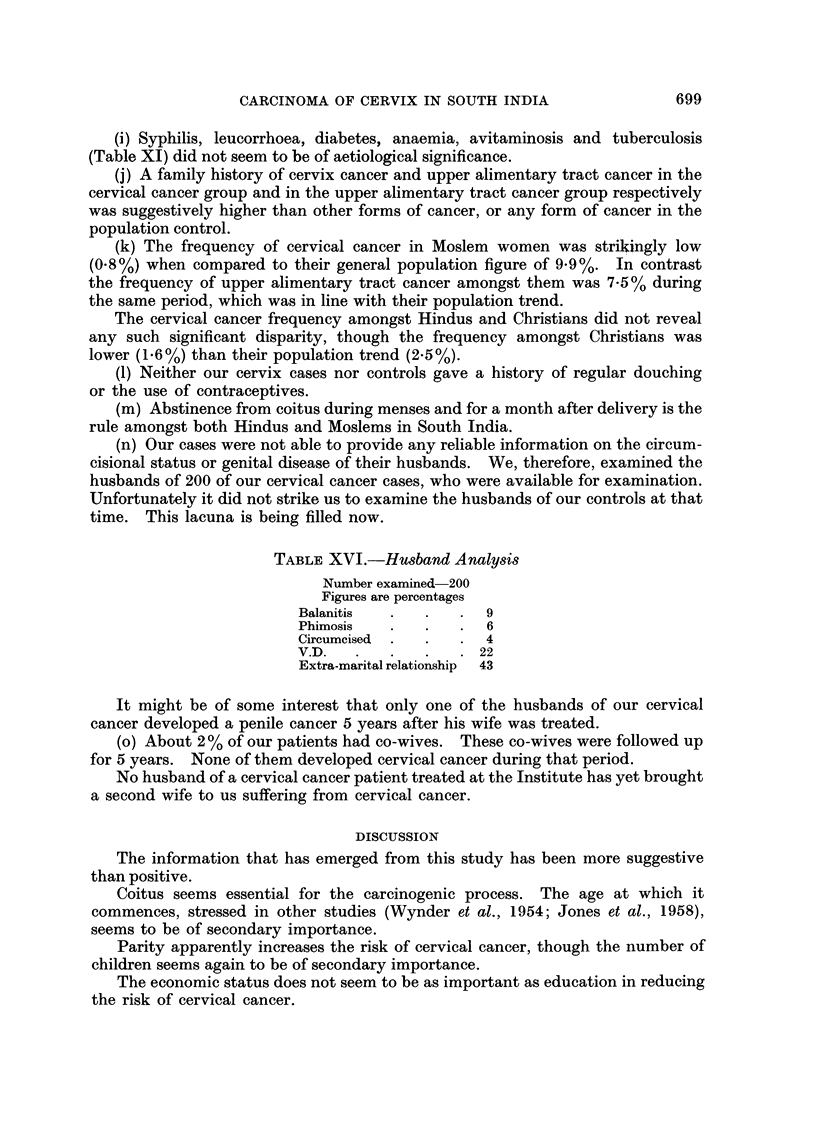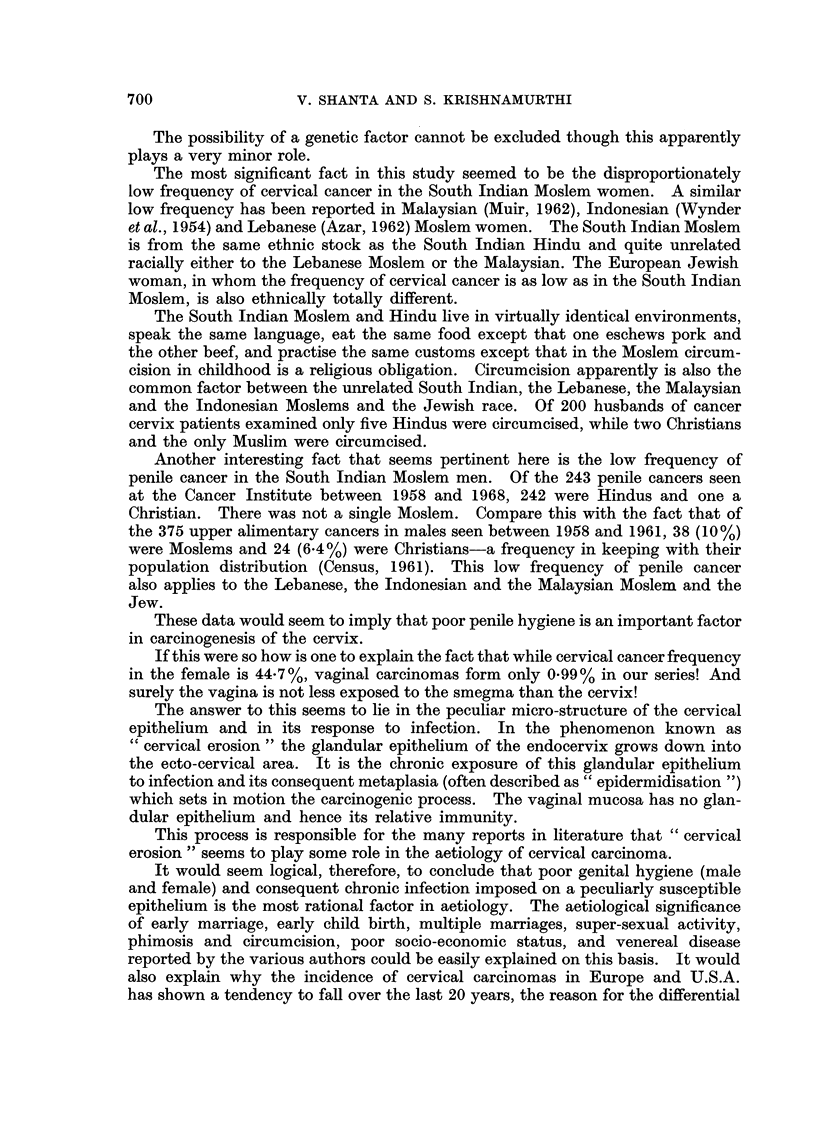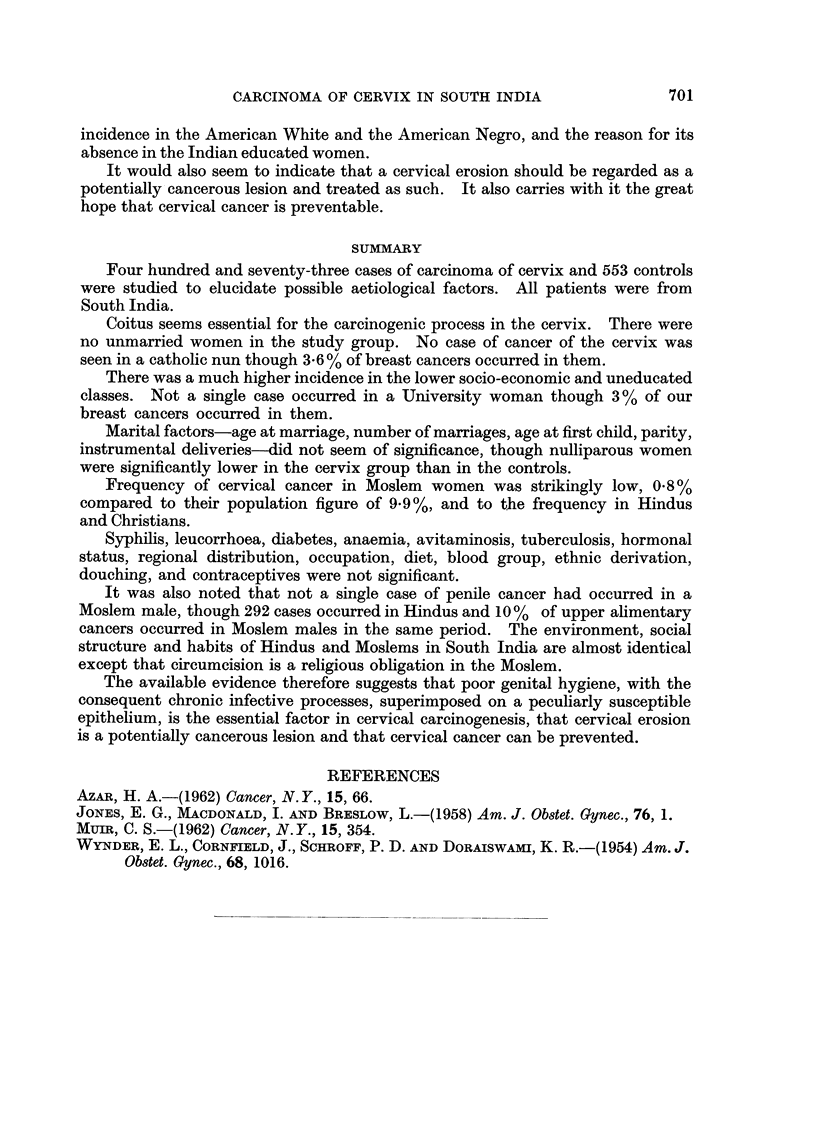# The aetiology of carcinoma of the uterine cervix in South India: a preliminary report.

**DOI:** 10.1038/bjc.1969.85

**Published:** 1969-12

**Authors:** V. Shanta, S. Krishnamurth


### THE AETIOLOGY OF CARCINOMA OF THE UTERINE CERVIX IN SOUTH INDIA: A PRELIMINARY REPORT

V. SHANTA AND S. KRISHNAMURTHI

*From the Cancer Institute, Madras, India*

Received for publication August 30, 1969

### MATERIAL

THIS preliminary report is based on a retrospective study of 473 cases of carcinoma cervix and 553 controls interviewed and examined during the period 1958–61. All the cervical cancers were in-patients at the Cancer Institute, Madras. Two hundred and fifty of the controls were drawn from an apparently healthy female population interviewed and examined during the rural surveys carried out by the Cancer Institute from time to time. A further 303 controls were drawn from women admitted to the Cancer Institute between 1958 and 1961 with carcinoma of the upper alimentary tract. All the cervical cancers and controls were consecutive cases admitted or seen during the period mentioned. No special attempt was made to match economic status or ethnic group or regional area or educational status. All the cervical cancers were squamous cell carcinomas.

### METHODS

The interviews were carried out by two trained medical social workers according to a prepared schedule. One of the interviewers was a male and the other a lady.

The cases and controls were examined personally by Dr. V. Shanta. Vaginal smear studies were carried out in every case. All the cancers were proved by biopsy. All the controls with the slightest suspicion of an unhealthy cervix or endometrial pathology were biopsied to exclude any pre-cancerous dysplasias or carcinomas *in situ*. All the smears and tissues were reported from our Pathology Laboratory, and all haematological and biochemical investigations were carried out in our clinical pathology and biochemical laboratories.

The factors studied were age, religion, ethnic group, regional (rural or urban) distribution, educational status, socio-economic status, menstrual history (menarche, rhythm, and menopause), marital history (age at marriage, widowhood, parity, abortions, medical attendance during labour), pre-existing pathology, family history, occupation, diet, blood group, genital hygiene (including that of the husband), circumcisional status of the husband, gynaecological pathology in co-wives, if any, and miscellaneous factors (contraception, urinary oestrogen excretion, etc.).

The findings are conveniently shown by a series of Tables.

TABLE I.—*Age*

(Figures are percentages)

| Decade | Carcinoma cervix | Controls: Hospital (U.A.C.)* | Controls: Population (healthy) |
|---|---|---|---|
| 20–29 yrs. | 14 | 5 | 45 |
| 30–39 „ | 103 | 40 | 69 |
| 40–49 „ | 166 | 91 | 75 |
| 50–59 „ | 139 | 102 | 42 |
| 60–69 „ | 43 | 55 | 17 |
| 70 & over | 8 | 10 | 2 |
| Total | 473 | 303 | 250 |

TABLE II—*Age Range*

(Figures are percentages)

| Age incidence in years | Carcinoma cervix (473) | Controls: Hospital (U.A.C.)* (303) | Controls: Population (healthy) (250) |
|---|---|---|---|
| Mean age | 45·3 | 40·6 | 48·9 |
| Youngest | 23 | 28 | 20 |
| Oldest | 78 | 72 | 70 |

* U.A.C. Upper alimentary carcinoma.

TABLE III.—*Religion*

(Figures are percentages)

| Religion | Carcinoma cervix (473) | Control: Hospital (U.A.C.) (303) | Control: Population (healthy) (250) | Ind. census 1961 |
|---|---|---|---|---|
| Hindu | 97·4 | 89·9 | 98·4 | 87·2 |
| Moslem | 0·8 | 7·5 | 0·8 | 9·9 |
| Christian | 1·6 | 2·6 | 0·8 | 2·5 |
| Others | 0·2 | nil | nil | 0·4 |

TABLE IV.—*Ethnic Group*

(Figures are percentages)

| Ethnic derivation | Carcinoma cervix (473) | Controls hospital (U.A.C.) (303) |
|---|---|---|
| Tamil | 72·3 | 86·6 |
| Andhra | 19·1 | 14·4 |
| Malayalee | 5·4 | 2·4 |
| Others | 3·2 | 0·6 |

TABLE V.—*Region*

(Figures are percentages)

| | Carcinoma cervix (473) | Controls hospital (U.A.C.) (303) | Ind. census 1961 |
|---|---|---|---|
| Rural | 64·4 | 56·2 | 75·6 |
| Urban | 35·6 | 43·8 | 24·4 |

### Table VI.—*Education*

(Figures are percentages)

| Standard | Carcinoma cervix (473) | Controls: Hospital (U.A.C.) (303) | Controls: Population (healthy) (250) | Ind. Census 1961 |
|---|---|---|---|---|
| Illiterate | 90·2 | 92·4 | 81·2 | 90·5 |
| School | 9·8 | 7·6 | 18·8 | 7·63 |
| University | nil | nil | nil | 2·32 |

### Table VII.—*Socio-Economic Status*

(Figures are percentages)

| Class | Carcinoma cervix (473) | Controls hospital (U.A.C.) (303) |
|---|---|---|
| Labour | 29·2 | 39·6 |
| Lower middle class | 63·2 | 52·8 |
| Upper middle class | 7·6 | 7·6 |

### Table VIII.—*Menarche*

(Figures are percentages)

| Age (years) | Carcinoma cervix (473) | Controls: Hospital (U.A.C.) (303) | Controls: Population (healthy) (250) |
|---|---|---|---|
| 11 | 4·4 | 1·3 | 3·2 |
| 12 | 17·75 | 13·4 | 14·0 |
| 13 | 23·8 | 25·5 | 25·0 |
| 14 | 27·0 | 28·5 | 27·0 |
| 15 | 16·9 | 23·2 | 23·0 |
| 16 | 5·0 | 6·2 | 5·0 |
| Over 16 | 4·0 | 4·2 | 1·0 |
| Not known | 1·0 | nil | nil |

### Table IX (*a*).—*Menstrual History*

(Figures are percentages)

| Menstruation | Carcinoma cervix (473) | Controls: Hospital (U.A.C.) (303) | Controls: Population (healthy) (250) |
|---|---|---|---|
| Menarche (mean age) | 13·4 years | 13·9 years | 13·7 years |
| Normal | 95·8 | 98·4 | 87·2 |
| Irregular (excess, prolonged or frequent) | 3·7 | 0·6 | 11·2 |
| Scanty | 0·5 | 1·0 | 1·6 |

### Table IX (*b*).—*Menstrual History*

(Figures are percentages)

| | Carcinoma cervix (473) | Controls: Hospital (U.A.C.) (303) | Controls: Population (healthy) (250) |
|---|---|---|---|
| Number menopaused | 49·5 | 62·1 | 36 |
| Mean age at menopause | — | — | — |
| Duration of menstrual life | — | — | — |

### Table X (*a*).—*Marital History*

(Figures are percentages)

| History | Carcinoma cervix (473) | Controls: Hospital (U.A.C.) (303) | Controls: Population (healthy) (250) |
|---|---|---|---|
| Unmarried (no sex relationships) | nil | 0·2 | nil |
| Married with age at marriage | | | |
| Below 10 years | 6·3 | 4·8 | 2·0 |
| 10–15 years | 55·2 | 61·0 | 65·6 |
| 16–20 years | 32·8 | 27·5 | 31·2 |
| Over 20 years | 4·6 | 6·5 | 1·2 |
| Details not known | 1·1 | nil | nil |
| Widows | 13·5 | 33·9 | 12·4 |

### Table X (*b*).—*Marital History*

(Figures are percentages)

| Parity | Carcinoma cervix (473) | Controls: Hospital (U.A.C.) (303) | Controls: Population (healthy) (250) |
|---|---|---|---|
| 0 | 4·4 | 11·5 | 11·6 |
| 1 | 6·9 | 10·8 | 7·6 |
| 2 | 7·6 | 10·5 | 12·4 |
| 3 | 10·7 | 11·8 | 10·1 |
| 4 | 15·0 | 13·2 | 15·2 |
| 5 | 14·3 | 7·2 | 9·6 |
| 6 | 9·3 | 11·1 | 12·0 |
| 7 | 9·0 | 6·9 | 7·2 |
| 8 | 9·9 | 6·6 | 5·2 |
| 9 | 5·4 | 2·1 | 3·6 |
| 10 | 3·8 | 3·3 | 3·7 |
| Over 10 | 3·1 | 3·0 | 2·4 |
| Mean number of children | 5 | 3·7 | 4·2 |
| Abortions | 20·8 | 12·1 | 24·0 |

### Table X (*c*).—*Marital History*

(Figures are percentages)

| Medical attention during labour | Carcinoma cervix (473) | Controls: Hospital (U.A.C.) (303) | Controls: Population (healthy) (250) |
|---|---|---|---|
| Yes | 1·8 | 5·2 | 9·0 |
| No | 98·2 | 94·8 | 91·0 |
| Forceps delivery | 2·6 | 0 | 0 |
| Caesarean | 0·2 | 0 | 0 |

TABLE XI.—*Pre-existing Disease or Pathology*

(Figures are percentages)

| Disease | Carcinoma cervix (473) | Controls: Hospital (U.A.C.) (303) | Controls: Population (healthy) (250) |
|---|---|---|---|
| Syphilis (Serology +ve) | 4 | 4·9 | 5·2 |
| Leucorrhoea | 9·5 | 10·6 | 58 |
| Diabetes mellitus (Glycosuria) | 2·3 | 0 | 2·8 |
| Anaemia (Hb less than 60% Sahli) | 28·1 | 15·8 | 64·0 |
| Avitaminosis | 0·6 | 5·6 | 36·0 |
| Pulmonary tuberculosis | 0·2 | 0 | 0 |

TABLE XII.—*Family History of Cancer*

(Figures are percentages)

| Family History | Carcinoma cervix (473) | Controls: Hospital (U.A.C.) (303) | Controls: Population (healthy) (250) |
|---|---|---|---|
| Cervix | 2·3 | 0 | 0 |
| U.A.C. | 0·2 | 1·7 | 0 |
| Stomach | 0·6 | 0·3 | 0 |
| Breast | 0·2 | 0 | 0 |

TABLE XIII.—*Occupation*

(Figures are percentages)

| Occupation | Carcinoma cervix (473) | Controls: Hospital (U.A.C.) (303) | Controls: Population (healthy) (250) |
|---|---|---|---|
| Housewife | 96·1 | 78·5 | 54 |
| Unskilled labour | 3·1 | 15·0 | 26·8 |
| Petty shopkeeper | 0·2 | 1·5 | 1·2 |
| Industrial labour | 0·2 | 0 | 0 |
| Weaver | 0 | 0·3 | 17·5 |
| Teacher | 0·4 | 2·6 | 0 |
| Miscellaneous | 0 | 2·1 | 0·5 |

TABLE XIV.—*Diet*

(Figures are percentages)

| Diet | Carcinoma cervix (473) | Controls: Hospital (U.A.C.) (303) | Controls: Population (healthy) (250) |
|---|---|---|---|
| Vegetarian | 23·6 | 22·7 | 19 |
| Non-vegetarian | 76·4 | 77·3 | 81 |

TABLE XV.—*Blood Groups*
(Figures are percentages)

| Blood groups | Carcinoma cervix (473) | Controls: Hospital (U.A.C.) (303) | Controls: Population healthy) (250) |
|---|---|---|---|
| A | 24·5 | 24·9 | 17·7 |
| B | 29·7 | 33·7 | 37·8 |
| O | 35·6 | 35·0 | 40·0 |
| AB | 10·2 | 6·4 | 4·5 |

### COMMENTARY

(a) The study group and the controls were nearly matched as regards age distribution.

(b) The large number of Tamils in the series is because the Institute is situated in the Tamil region and does not signify any special ethnic predisposition.

(c) Occupation, diet and blood groups did not seem of any aetiological significance.

(d) There was a tendency for a higher urban incidence than the general population distribution would admit.

(e) As in most other reported series the lower socio-economic classes and the uneducated were the most affected. But this incidence in the lower and ignorant classes is the same in the cervix group and the control group and also follows generally the socio-economic distribution of the population at large. Its significance, therefore, as an aetiological factor is a matter for doubt.

It is, however, significant that we have never come across a carcinoma cervix in a University educated woman, though 3% of our carcinoma of the breast patients have been from that category.

(f) Our urinary oestrogen assays seemed to us unreliable. The menstrual history (Tables VIII and IX) did not reveal any significant differences between the cervix group and the controls.

(g) Marital history did not reveal any significant difference between the cervix and the control groups in regard to spinsterhood, age at marriage or widowhood. There was no case of divorce in our series, though a few women were deserted after they developed the cervical cancer. No history of extra-marital relationship was given by the women either in the cervix or control groups though it seemed to be the rule amongst the men. Hindu tradition is such that infidelity amongst Hindu women is very rare.

94·3% of the cervical cancers, 93·3% of the hospital controls and 98·8% of the general population controls were married before the age of 20 years.

It might, however, be significant that we have not seen a single case of carcinoma cervix in a Catholic nun, though 3·6% of our breast cancers were drawn from them.

(h) The number of children or the number of abortions were not significantly different between the cervix and the control groups, though the percentage of nulliparous women was significantly lower in the cervix group than in the controls.

Medical attendance during labour was slightly lower and the number of instrumental deliveries slightly higher in the cervix group than in the controls.

In 92% of the cervical cancers, 86% of the hospital controls and 88% of the population controls the first child was born before the age of 20 years. The age at which the last child was born was not recorded.

(i) Syphilis, leucorrhoea, diabetes, anaemia, avitaminosis and tuberculosis (Table XI) did not seem to be of aetiological significance.

(j) A family history of cervix cancer and upper alimentary tract cancer in the cervical cancer group and in the upper alimentary tract cancer group respectively was suggestively higher than other forms of cancer, or any form of cancer in the population control.

(k) The frequency of cervical cancer in Moslem women was strikingly low (0·8%) when compared to their general population figure of 9·9%. In contrast the frequency of upper alimentary tract cancer amongst them was 7·5% during the same period, which was in line with their population trend.

The cervical cancer frequency amongst Hindus and Christians did not reveal any such significant disparity, though the frequency amongst Christians was lower (1·6%) than their population trend (2·5%).

(l) Neither our cervix cases nor controls gave a history of regular douching or the use of contraceptives.

(m) Abstinence from coitus during menses and for a month after delivery is the rule amongst both Hindus and Moslems in South India.

(n) Our cases were not able to provide any reliable information on the circumcisional status or genital disease of their husbands. We, therefore, examined the husbands of 200 of our cervical cancer cases, who were available for examination. Unfortunately it did not strike us to examine the husbands of our controls at that time. This lacuna is being filled now.

TABLE XVI.—*Husband Analysis*

Number examined—200
Figures are percentages

| | |
|---|---|
| Balanitis | 9 |
| Phimosis | 6 |
| Circumcised | 4 |
| V.D. | 22 |
| Extra-marital relationship | 43 |

It might be of some interest that only one of the husbands of our cervical cancer developed a penile cancer 5 years after his wife was treated.

(o) About 2% of our patients had co-wives. These co-wives were followed up for 5 years. None of them developed cervical cancer during that period.

No husband of a cervical cancer patient treated at the Institute has yet brought a second wife to us suffering from cervical cancer.

### DISCUSSION

The information that has emerged from this study has been more suggestive than positive.

Coitus seems essential for the carcinogenic process. The age at which it commences, stressed in other studies (Wynder *et al.*, 1954; Jones *et al.*, 1958), seems to be of secondary importance.

Parity apparently increases the risk of cervical cancer, though the number of children seems again to be of secondary importance.

The economic status does not seem to be as important as education in reducing the risk of cervical cancer.

The possibility of a genetic factor cannot be excluded though this apparently plays a very minor role.

The most significant fact in this study seemed to be the disproportionately low frequency of cervical cancer in the South Indian Moslem women. A similar low frequency has been reported in Malaysian (Muir, 1962), Indonesian (Wynder *et al.*, 1954) and Lebanese (Azar, 1962) Moslem women. The South Indian Moslem is from the same ethnic stock as the South Indian Hindu and quite unrelated racially either to the Lebanese Moslem or the Malaysian. The European Jewish woman, in whom the frequency of cervical cancer is as low as in the South Indian Moslem, is also ethnically totally different.

The South Indian Moslem and Hindu live in virtually identical environments, speak the same language, eat the same food except that one eschews pork and the other beef, and practise the same customs except that in the Moslem circumcision in childhood is a religious obligation. Circumcision apparently is also the common factor between the unrelated South Indian, the Lebanese, the Malaysian and the Indonesian Moslems and the Jewish race. Of 200 husbands of cancer cervix patients examined only five Hindus were circumcised, while two Christians and the only Muslim were circumcised.

Another interesting fact that seems pertinent here is the low frequency of penile cancer in the South Indian Moslem men. Of the 243 penile cancers seen at the Cancer Institute between 1958 and 1968, 242 were Hindus and one a Christian. There was not a single Moslem. Compare this with the fact that of the 375 upper alimentary cancers in males seen between 1958 and 1961, 38 (10%) were Moslems and 24 (6·4%) were Christians—a frequency in keeping with their population distribution (Census, 1961). This low frequency of penile cancer also applies to the Lebanese, the Indonesian and the Malaysian Moslem and the Jew.

These data would seem to imply that poor penile hygiene is an important factor in carcinogenesis of the cervix.

If this were so how is one to explain the fact that while cervical cancer frequency in the female is 44·7%, vaginal carcinomas form only 0·99% in our series! And surely the vagina is not less exposed to the smegma than the cervix!

The answer to this seems to lie in the peculiar micro-structure of the cervical epithelium and in its response to infection. In the phenomenon known as " cervical erosion " the glandular epithelium of the endocervix grows down into the ecto-cervical area. It is the chronic exposure of this glandular epithelium to infection and its consequent metaplasia (often described as " epidermidisation ") which sets in motion the carcinogenic process. The vaginal mucosa has no glandular epithelium and hence its relative immunity.

This process is responsible for the many reports in literature that " cervical erosion " seems to play some role in the aetiology of cervical carcinoma.

It would seem logical, therefore, to conclude that poor genital hygiene (male and female) and consequent chronic infection imposed on a peculiarly susceptible epithelium is the most rational factor in aetiology. The aetiological significance of early marriage, early child birth, multiple marriages, super-sexual activity, phimosis and circumcision, poor socio-economic status, and venereal disease reported by the various authors could be easily explained on this basis. It would also explain why the incidence of cervical carcinomas in Europe and U.S.A. has shown a tendency to fall over the last 20 years, the reason for the differential incidence in the American White and the American Negro, and the reason for its absence in the Indian educated women.

It would also seem to indicate that a cervical erosion should be regarded as a potentially cancerous lesion and treated as such. It also carries with it the great hope that cervical cancer is preventable.

SUMMARY

Four hundred and seventy-three cases of carcinoma of cervix and 553 controls were studied to elucidate possible aetiological factors. All patients were from South India.

Coitus seems essential for the carcinogenic process in the cervix. There were no unmarried women in the study group. No case of cancer of the cervix was seen in a catholic nun though 3·6% of breast cancers occurred in them.

There was a much higher incidence in the lower socio-economic and uneducated classes. Not a single case occurred in a University woman though 3% of our breast cancers occurred in them.

Marital factors—age at marriage, number of marriages, age at first child, parity, instrumental deliveries—did not seem of significance, though nulliparous women were significantly lower in the cervix group than in the controls.

Frequency of cervical cancer in Moslem women was strikingly low, 0·8% compared to their population figure of 9·9%, and to the frequency in Hindus and Christians.

Syphilis, leucorrhoea, diabetes, anaemia, avitaminosis, tuberculosis, hormonal status, regional distribution, occupation, diet, blood group, ethnic derivation, douching, and contraceptives were not significant.

It was also noted that not a single case of penile cancer had occurred in a Moslem male, though 292 cases occurred in Hindus and 10% of upper alimentary cancers occurred in Moslem males in the same period. The environment, social structure and habits of Hindus and Moslems in South India are almost identical except that circumcision is a religious obligation in the Moslem.

The available evidence therefore suggests that poor genital hygiene, with the consequent chronic infective processes, superimposed on a peculiarly susceptible epithelium, is the essential factor in cervical carcinogenesis, that cervical erosion is a potentially cancerous lesion and that cervical cancer can be prevented.

### REFERENCES

Azar, H. A.—(1962) *Cancer, N.Y.*, **15**, 66.

Jones, E. G., Macdonald, I. and Breslow, L.—(1958) *Am. J. Obstet. Gynec.*, **76**, 1.

Muir, C. S.—(1962) *Cancer, N.Y.*, **15**, 354.

Wynder, E. L., Cornfield, J., Schroff, P. D. and Doraiswami, K. R.—(1954) *Am. J. Obstet. Gynec.*, **68**, 1016.